# Supplementary material for: Multimodal AI agents for capturing and sharing proteomics laboratory practice
Source: Mol Syst Biol. 2025 Dec 15;22(3):333–6. doi: 10.1038/s44320-025-00179-1 (PMC12954122; doi:10.1038/s44320-025-00179-1)
Supplement: Supplementary file 1 — Appendix [file 44320_2025_179_MOESM1_ESM.docx]

Appendix for “Multimodal AI agents for capturing and sharing proteomics laboratory practice”

This Appendix contains additional figures and extended methods.

Table of Contents

[Appendix figures 1](#_Toc215303275)

[Extended methods 5](#_Toc215303276)

[Agent framework 5](#_Toc215303277)

[Agent workflow 6](#_Toc215303278)

[Scenario A: Instrument performance queries 6](#_Toc215303279)

[Scenario B: Automatic protocol generation 6](#_Toc215303280)

[Scenario C: Video analysis to detect errors 7](#_Toc215303281)

[Agent benchmarking 8](#_Toc215303282)

[Benchmarking of protocol generation 8](#_Toc215303283)

[Benchmarking of error detection 13](#_Toc215303284)

[References 16](#_Toc215303285)

# Appendix figures

[***Appendix Figure S1: Agentic workflow for instrument performance queries.*** 2](#_Toc215303287)

[***Appendix Figure S2: Agentic workflow for automatic protocol generation.*** 2](#_Toc215303288)

[***Appendix Figure S3: Performance metrics for the protocol generation task.*** 3](#_Toc215303289)

[***Appendix Figure S4: Agentic workflow for error detection and lab note generation.*** 4](#_Toc215303290)

[***Appendix Figure S5: Performance of error detection and lab notes.*** 5](#_Toc215303291)


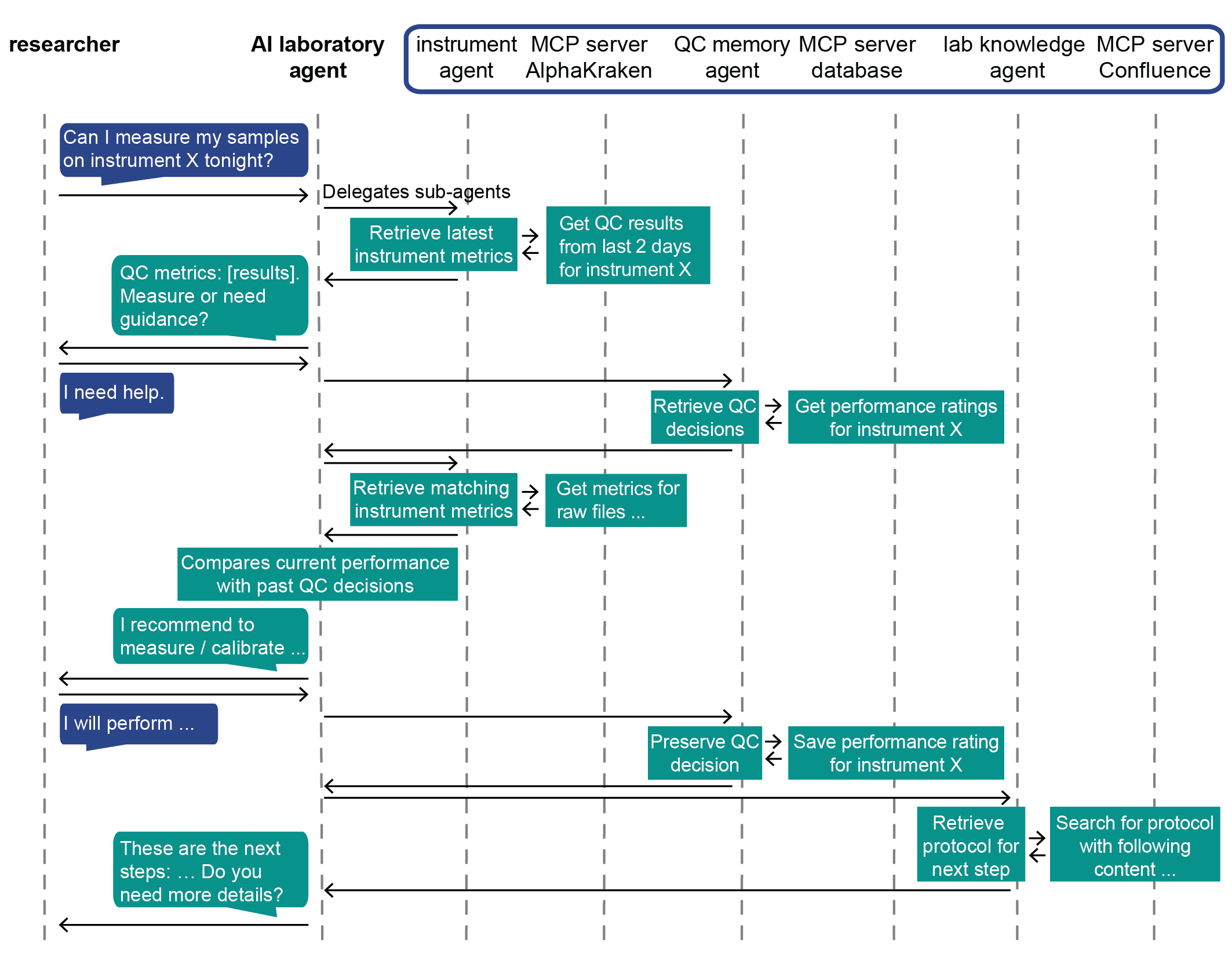


***Appendix Figure S1: Agentic workflow for instrument performance queries.***

*The flow of information between the user, the Main Agent, and the specialized sub-agents (Instrument, QC Memory, Lab Knowledge) during an instrument performance query.*


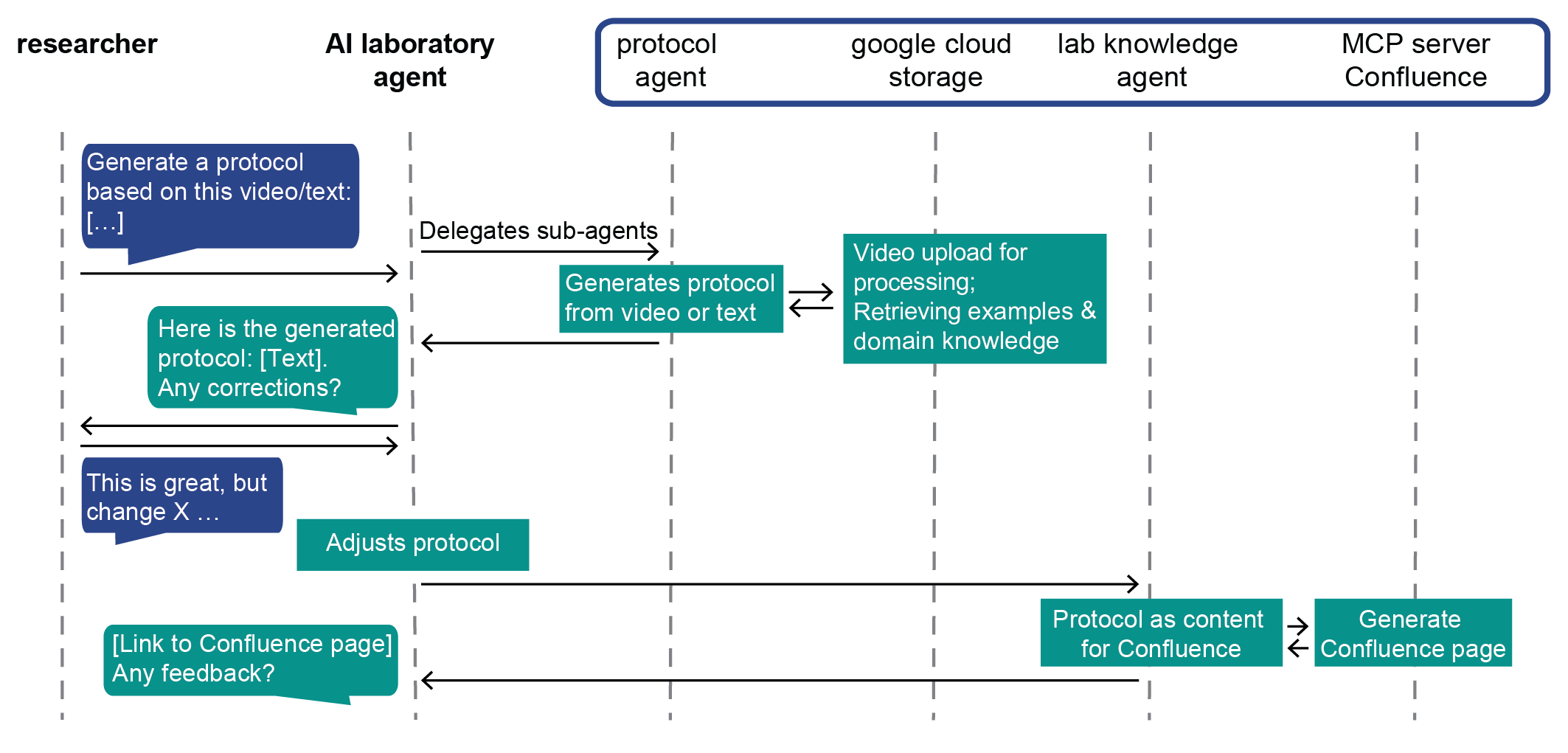


***Appendix Figure S2: Agentic workflow for automatic protocol generation.***

*The process from video upload to the final, user-approved protocol stored on a documentation platform such as Confluence.*

***
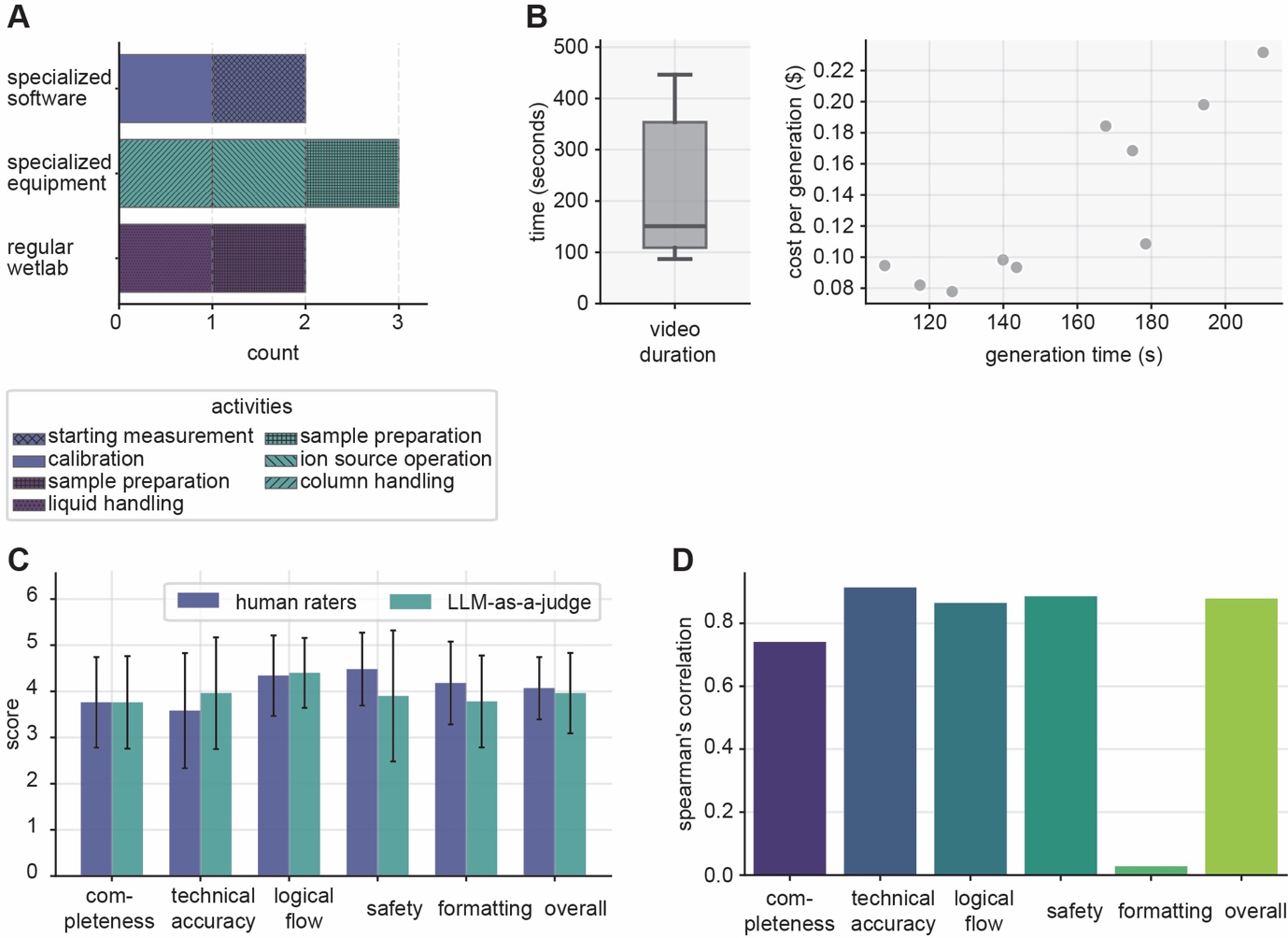
***

***Appendix Figure S3: Performance metrics for the protocol generation task.***

***(A)*** *Composition of the benchmark dataset, categorized into specialized software, specialized equipment, and regular wet-lab protocols.* ***(B)*** *The left panel shows a box plot of the source video durations. The right scatter plot illustrates the relationship between the protocol generation time (in seconds) and the corresponding token cost per generation (in US dollars) for each protocol.* ***(C)*** *Evaluation scores for the generated protocols from five expert human raters (blue) and five LLM-as-a-judge iterations (green) across six categories: completeness, technical accuracy, logical flow, safety instructions, formatting, and an overall quality. The height of each bar represents the average score, and the error bars indicate the standard deviation.* ***(D)*** *Calibration of the "LLM-as-a-judge" against human ratings. Each bar displays the Spearman's rank correlation coefficient (ρ) for a single evaluation criterion. This coefficient quantifies the correlation between the mean scores of five expert human raters and the mean scores from the five LLM-as-a-judge iterations, calculated across the ten generated protocols.*


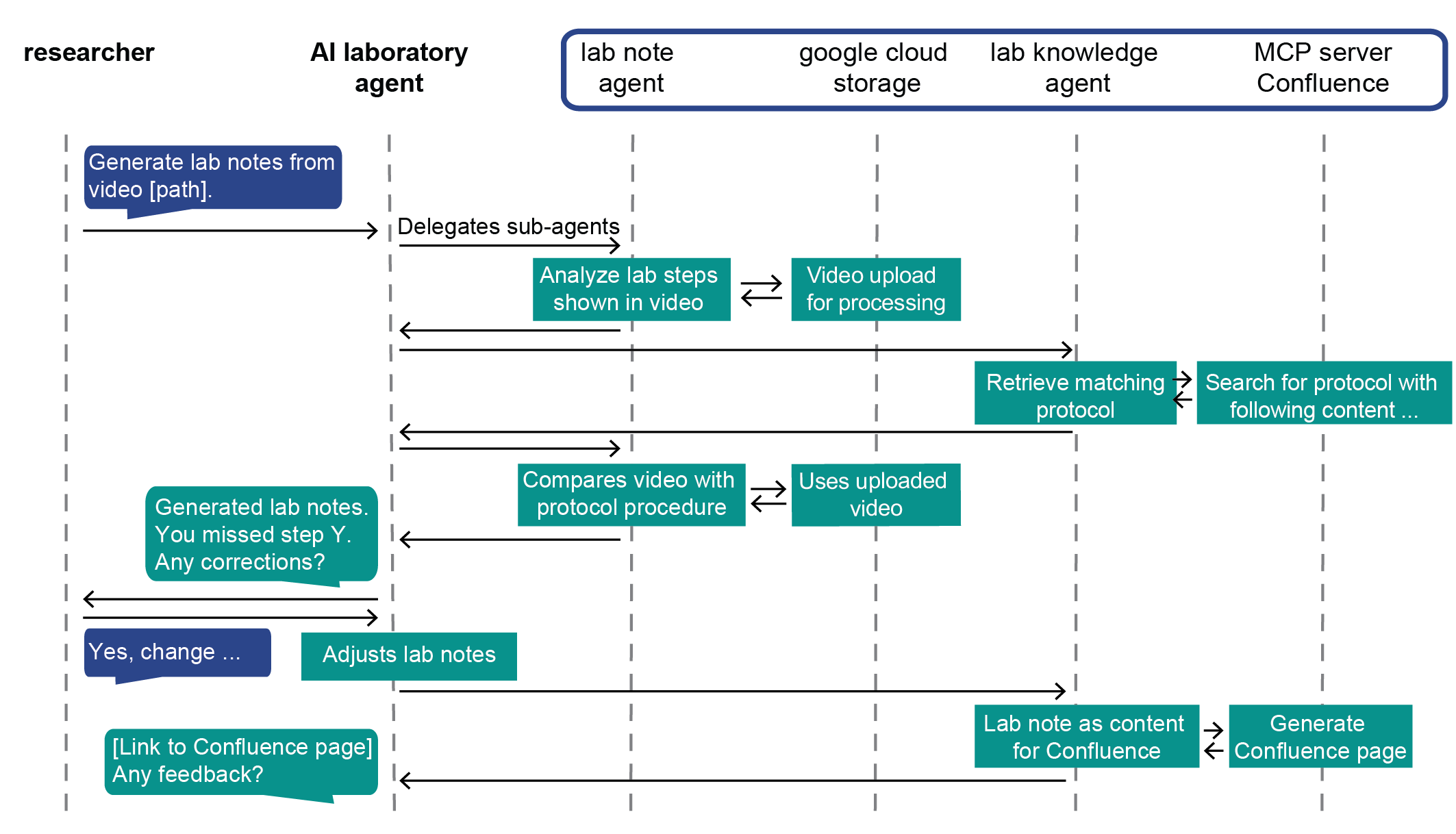


***Appendix Figure S4: Agentic workflow for error detection and lab note generation.***

*Illustration of the steps involved in identifying a protocol, comparing it against a video, flagging deviations, and generating final lab notes.*


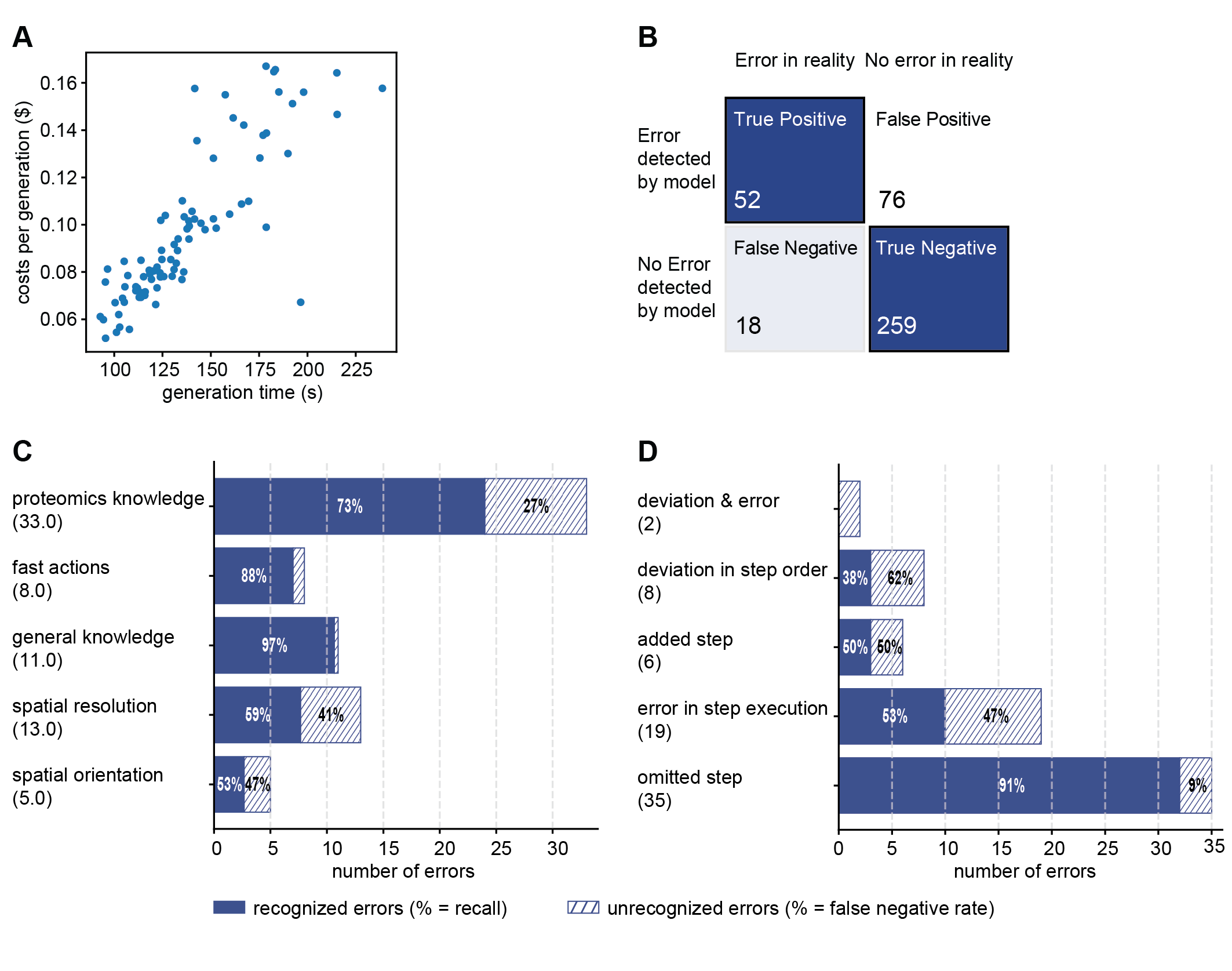


***Appendix Figure S5: Performance of error detection and lab notes.***

***(A)*** *Relationship between the generation time and the associated token cost (in US dollars) for each lab note created.* *Lab notes were generated from each video in triplicate.* ***(B)*** *Confusion matrix summarizing the model's error detection performance on the benchmark dataset.****(C)*** *Errors categorized by the underlying AI skill limitation: Domain-specific proteomics knowledge, detection of fast actions, spatial resolution (e.g., reading the volume on a pipette) or spatial orientation (e.g., exact tip position in a rack). The numbers in parentheses indicate the total number of introduced errors for each category.* ***(D)*** *Error recognition by the type of protocol deviation.*

# Extended methods

## Agent framework

The system is a multi-agent AI framework designed to provide laboratory guidance and capture institutional knowledge. It is built on Google's Agent Development Kit (ADK) and uses Google's Gemini models as its foundation. Specifically, Gemini 2.5 Flash was used for the agent's main functionalities, while Gemini 2.5 Pro was used for more complex video analysis. The agent accesses external data sources and tools such as Confluence pages via the Model Context Protocol (MCP). For video analysis tasks, a custom proteomics knowledge base and video files were retrieved from Google Cloud Storage and provided directly to the prompt.

All agent actions are directed by a Main Agent that functions as an orchestrator. It receives user requests from a chat interface, interprets them, and delegates tasks to a team of specialized sub-agents:

- Protocol Agent: Generates formatted, publication-ready protocols by analyzing video recordings of laboratory procedures (see below for more details).
- Lab Note Agent: Compares a video of a researcher executing a protocol against a baseline text protocol to detect errors, omissions, or other deviations. Subsequently, the agent generates a laboratory note flagging the found errors.
- Lab Knowledge Agent: Manages interactions with an internal knowledge base hosted on Confluence. In our implementation, the agent uses a local MCP server ([mcp-atlassian](https://github.com/sooperset/mcp-atlassian)) to search, retrieve, and generate Confluence pages.
- Instrument Agent: Retrieves current and historical performance metrics for mass spectrometers via a local [AlphaKraken](https://github.com/MannLabs/alphakraken) MCP server. AlphaKraken is an in-house developed tool for automated data processing and monitoring of mass spectrometry experiments.
- QC Memory Agent: Logs quality control (QC) decisions and user ratings to a local SQL database, accessed via an MCP server, to create a persistent history of instrument operation and troubleshooting.

## Agent workflow

The role of the Main Agent is to be an AI research assistant with broad knowledge of proteomics. Its core logic matches user requests to predefined scenarios.

### Scenario A: Instrument performance queries

This workflow is triggered when a user asks about instrument availability or performance (e.g., "Can I run my sample on tims1 tonight?", where tims1 is one of the mass spectrometers). The agent follows a multi-step process (see Appendix Figure S1 above):

1. First, the Main Agent invokes the Instrument Agent to retrieve the latest QC metrics from the AlphaKraken server.
2. The user is prompted to make a decision based on the data provided by the Agent (e.g., "Would you proceed with measuring or do you need guidance?").
3. If guidance is requested, the QC Memory Agent retrieves historical decisions made under similar QC conditions (same instrument and analytical gradient) from the SQL database. This database stores the decision (measure: yes/no), a performance rating (1-5), and a comment linked to the specific raw file name, instrument, and gradient. The remaining QC metrics of each raw file are again retrieved with the AlphaKraken MCP server.
4. The agent synthesizes the current and historical data to provide an actionable recommendation (e.g., “Other researchers measured at this performance level,” or “You should calibrate the TOF analyzer.”).
5. After the user decides on a course of action, the QC Memory Agent logs this decision, preserving it as institutional memory.
6. Finally, the Lab Knowledge Agent retrieves relevant protocols for the user's next steps.

### Scenario B: Automatic protocol generation

This workflow is triggered when a user requests to create a protocol from a video file or text notes (Appendix Figure S2). This requires a researcher to have recorded how they executed a procedure while explaining it, in order to create a tutorial video or notes about that procedure.

1. The Main Agent triggers the Protocol Agent, which uploads the video to Google Cloud Storage and then analyzes its visual and audio streams to produce a formatted, publication-ready protocol. Alternatively, one can use the notes as input.
2. The user can revise and approve the generated protocol through the chat interface.
3. Once the user is satisfied, the Main Agent invokes the Lab Knowledge Agent to save the final protocol as a new page in Confluence.

To supplement the general-purpose LLM with proteomics expertise, we optimized the Protocol Agent instructions. This multi-part prompt begins by instructing the model to adopt an expert persona in the field (e.g., "You are Matthias Mann"). It then provides contextual knowledge through domain-specific background information, such as images of laboratory equipment and detailed text descriptions of these (e.g., "An UltraSource from Bruker looks like a black spheroid housing with a thick white corrugated tube attached"). Furthermore, the prompt includes a clear task definition, which specifies the output, for instance, as a "Nature-style" protocol with standardized sections like abstract, materials, a step-by-step procedure, and expected results.

To guide its reasoning, the prompt is formatted as chain-of-thought instructions, directing the model to first transcribe observations from the video with timestamps and then convert these observations into the structured protocol format. Finally, the prompt is grounded with few-shot examples, including a diverse set of complete video-protocol pairs that reflect possible user inputs, such as recordings from both a wearable camera and a screen. We found that our optimized prompt led to protocols that integrated both visual and verbal cues. In contrast, simple prompts (e.g., “Your task is to analyze the provided video and to convert it into a Nature-style protocol”) resulted in protocols that reflected primarily the spoken content.

### Scenario C: Video analysis to detect errors

This workflow is triggered when a researcher submits an experimental video with a prompt like, "Generate lab notes from this video and check for mistakes.” (Appendix Figure S4). This requires a researcher to record themselves while performing an experiment.

1. The Main Agent delegates the initial analysis to the Lab Note Agent, which uploads the video to Google Cloud Storage, followed by video analysis to generate a summary of the procedure performed.
2. The Main Agent then invokes the Lab Knowledge Agent to search the lab's Confluence for written protocols that match the video summary.
3. The agent suggests the best match to the user and asks for confirmation that this was the correct protocol.
4. Once confirmed, the Lab Knowledge Agent retrieves the full content of the correct protocol.
5. The Main Agent passes both the video and the baseline protocol to the Lab Note Agent for a detailed comparison. The agent systematically compares each step, flagging deviations such as omissions, additions, incorrect order, or procedural errors.
6. The agent outputs lab notes where potential errors are flagged for review.
7. After the researcher finalizes the notes, the Lab Knowledge Agent saves them to Confluence, preserving an accurate record of the experiment.

To detect errors during a lab procedure, we instructed the model to compare a written protocol with a video of the experiment. Our prompt directs the model to act as an expert, “Professor Matthias Mann”, and provides it with background documents on proteomics. The main task is to compare the video and the protocol, marking each step in the written protocol with a specific label: Correct, Omitted, Error, Added, or Deviation. This task follows an optimized chain-of-thought logic. The model begins by understanding the original written protocol and then goes through the video to create a timestamped log of all actions and spoken words. Next, it matches each event in the video log to the corresponding step in the protocol. For every potential mismatch it finds, the model re-examines the relevant video segment. After confirming all differences, the model generates the final annotated lab notes. The prompt also includes one complete example - a written protocol, a video, and the correct final output – to guide the task execution.

Generating lab notes includes a series of binary error detection decisions (i.e., flagging a step as 'correct' or 'wrong'). To ensure the consistency of these decisions, we selected a low sampling temperature of 0.2. While a temperature of 0.0 would offer complete determinism, it is not ideal for this generative task, which involves multiple tokens. In our experience, a temperature of 0.0 occasionally caused the model to enter undesirable generation loops (e.g., repeating the same phrase or word multiple times), resulting in an incomplete or malformed lab note. A low temperature of 0.2 provides the optimal balance: it is sufficiently low to make the error detection consistent and reproducible, while introducing just enough stochasticity to prevent these repetitive generative artifacts.

## Agent benchmarking

### Benchmarking of protocol generation

We compiled a benchmark dataset of ten protocol recordings with a wearable camera (GoPro HERO8 black) covering a range of activities, from standard wet-lab procedures (e.g., pipetting) to interactions with specialized equipment (e.g., modifying MS ion sources) and software (e.g., instrument calibration) (Appendix Figure S3 A). These protocols were adapted from our recently published Nature Protocol about operating the mass spectrometer timsTOF from Bruker featuring the use of IonOpticks columns, Evotips that contain samples, and Evosep as a liquid chromatography system (Skowronek *et al*, 2025).

For automated evaluation, we used an ‘LLM-as-a-judge’ approach (Zheng *et al*, 2023), where a separate model compared the AI-generated protocols with manually created ground-truth section by section and step by step. To ensure a general-purpose evaluation applicable to protocols from diverse domains, the LLM-as-a-judge prompt first instructs the model to adopt a domain-independent persona as an "expert evaluator". It then follows a structured reasoning process, directing the model to first read both the ground truth and AI-generated protocols and systematically compare each section (e.g., Title, Abstract, Materials, Procedure steps). Finally, based on this detailed comparison, the LLM is instructed to provide a single, holistic rating for each protocol on a 1-5 (5: very good) scale across the five evaluation criteria (see Table 1). To calculate the final overall quality score, the scores of the five evaluation criteria were averaged.

To validate this 'LLM-as-a-judge' approach, we performed a calibration experiment. Five expert human raters were tasked with evaluating the same ten AI-generated protocols against the ground-truth protocols. Mirroring the LLM-judge, the human experts also provided a single, holistic score for each of the five evaluation criteria using the identical 1-5 rating rubric.

We found strong Spearman's correlations (ρ) for all critical scientific criteria, including Technical Accuracy (ρ≈0.91), Logical Flow (ρ≈0.86), Safety (ρ≈0.89), and Overall Quality (ρ≈0.88) (Appendix Figure S3 D). This analysis also revealed distinct rating profiles: human experts were more stringent on content-related criteria like Completeness and Technical Accuracy, which require expert understanding, whereas the LLM-judge was stricter when comparing the AI-protocol with the ground truth on Formatting (Appendix Figure S3 C). This divergence in focus explains the lack of correlation for the formatting rubric (ρ≈0.05), confirming the LLM and humans interpreted this specific criterion differently. Overall, this calibration experiment showed that there is a strong correlation between the LLM and human scores, confirming that our LLM judge is well-calibrated.

Table 1: Evaluation criteria.

| **Criterion** | **Definition** |
| --- | --- |
| Completeness | A comparison of what is present in both protocols, what is missing from the AI protocol, and what is extra in the AI protocol. |
| Technical Accuracy | Demonstrates scientific understanding by correctly distinguishing techniques/equipment and using appropriate terminology. |
| Logical Flow | The workflow maintains the chronological sequence of the procedure. |
| Safety | Appropriate identification and emphasis of critical cautions, warnings, and safety measures. |
| Formatting | The AI-generated protocol matches the formatting of the ground truth protocol. |

#### Example agent workflow video

<https://zenodo.org/records/17710382/files/ProtocolGeneration.mp4?download=1>

#### Example protocol video

<https://zenodo.org/records/17710382/files/ConnectingESISource_forProtocolGeneration.MP4?download=1>

#### Example ground truth protocol

**Installation of the ESI Source on a Bruker timsTOF SCP**

**Abstract**

This protocol provides a step-by-step guide for the correct installation of a Electrospray Ionization (ESI) source onto a Bruker timsTOF SCP mass spectrometer.

**Materials**

**Equipment**

- Bruker timsTOF SCP Mass Spectrometer
- ESI Source
- Capillary Cap
- Spray Shield
- Personal Protective Equipment (PPE): laboratory gloves
- Syringe filled with sample

**Reagents**

- Nitrogen gas supply (instrument-grade)

**Procedure**

Estimated timing: < 2 minutes

**Assemble the Ion Source Interface**

1. Put on a fresh pair of laboratory gloves to avoid contaminating the ion source components.
2. Carefully align the capillary cap with the glass capillary at the center of the instrument's ion source interface. Gently press it into place.

**CRITICAL STEP** Do not cover the central orifice of the cap while handling it, as this can disrupt the instrument's vacuum.

1. Place the conical spray shield over the installed capillary cap, ensuring it is seated correctly.

**Mount the ESI Source Door**

1. Pick up the spherical ESI source door and align its hinge with the corresponding mounting point on the mass spectrometer's chassis.
2. Swing the source door into its closed position against the interface.
3. Secure the door by fastening the metal locking latch until it is firmly engaged.

**Connect Gas and Sample Lines**

1. Connect the nitrogen gas line to the designated gas inlet port on the top of the ESI source.
2. Connect the sample capillary line to a syringe filled with sample, which then connects to the sample inlet port, typically located next to the gas port.

**Expected Results**

- The ESI source is securely mounted on the timsTOF SCP instrument front.
- All components (capillary cap, spray shield, source door) are correctly aligned and seated.
- The nitrogen gas and sample lines are firmly connected to their respective ports.
- The instrument maintains a stable vacuum, indicating a proper seal has been achieved, and is now ready for operation.

**Figures**

Figure 1: Ion Source Interface Components.

[Placeholder for an image showing the fully assembled source]

#### Example video analysis of agent

**1. Visible Objects and Equipment:**

- **Primary Equipment:** A Bruker timsTOF SCP mass spectrometer, characterized by its blue and black casing.
- **Ion Source Components:**
  - A black, spherical Electrospray Ionization (ESI) source door with warning symbols (high voltage, hot surface).
  - A small, ring-shaped, metallic component: the capillary cap.
  - A larger, conical, metallic component: the spray shield.
- **Connections:**
  - A blue plastic tube for the nitrogen gas supply.
  - A thin, reddish-brown capillary tube with a yellow finger-tight fitting for the sample line.
- **Researcher:** Hands wearing white, disposable laboratory gloves.
- **Workspace:** A typical mass spectrometry laboratory environment with the instrument in the center, a computer monitor to the left, and various power cords and lab benches in the background.

**2. Spatial Positions and Orientations:**

- The procedure is performed directly at the front of the timsTOF mass spectrometer, at the circular instrument inlet.
- The researcher is positioned directly in front of the instrument.
- The source components are initially held in the researcher's hands and then mounted sequentially onto the instrument interface.
- The nitrogen and sample lines are connected to the top of the ESI source door after it is mounted.

**3. Audible Information with Timestamps:**

- 0:01-0:04: "I want to connect the ESI source to the timsTOF." (Accompanied by the constant hum and hiss of the instrument).
- 0:06-0:15: "So for this, I first... add the capillary cap onto the capillary."
- 0:15-0:21: "Here it's important to not put the finger over the hole, otherwise you block the vacuum." (The sound of rushing air is audible, which lessens after the cap is placed).
- 0:21-0:24: "Then we are adding the spray shield."
- 0:25-0:32: "and hinge in the ESI source door. Let's close it."
- 0:33-0:38: "Let's connect nitrogen."
- 0:39-0:46: "and the sample line."
- 0:47-0:50: "This is how a well-connected source looks."

**4. Visible Actions with Timestamps:**

- 0:00-0:05: The camera shows the open source interface on the timsTOF, with the transfer capillary visible in the center.
- 0:13-0:18: The researcher, wearing gloves, carefully places the capillary cap over the instrument's transfer capillary. They use their thumb and index finger, avoiding a small hole on the cap's surface.
- 0:22-0:24: The conical spray shield is placed over the capillary cap, seating it onto the mounting plate.
- 0:26-0:30: The black, spherical ESI source door is aligned with the hinges on the instrument and mounted.
- 0:31-0:32: A silver lever on the right side of the source is pushed down to secure the source door.
- 0:34-0:38: The blue nitrogen gas line is firmly pushed into its corresponding port on top of the source door.
- 0:39-0:46: The sample line, ending in a yellow fitting, is screwed into the central port on the source door and finger-tightened.
- 0:47-0:52: The final assembly is shown, with all components correctly installed and connected.

#### Example ai-generated protocol

**Protocol for Installing the Electrospray Ionization (ESI) Source on a Bruker timsTOF Mass Spectrometer**

**Abstract**

This protocol details the step-by-step procedure for the correct installation of a standard Electrospray Ionization (ESI) source onto a Bruker timsTOF series mass spectrometer. The process involves the sequential assembly of the source interface components, mounting of the source housing, and connection of the necessary gas and sample lines for operation. Proper execution of this protocol is essential for establishing a stable spray and maintaining the instrument's high vacuum.

**Materials**

**Equipment**

- timsTOF series mass spectrometer (e.g., timsTOF SCP)
- Electrospray Ionization (ESI) source assembly, including:
- ESI source door (housing)
- Capillary cap
- Spray shield
- Nitrogen gas supply line
- Liquid chromatography (LC) system with sample delivery capillary

**Reagents**

- Nitrogen gas, high purity

**! CAUTION** Nitrogen is an asphyxiant in high concentrations. Ensure adequate laboratory ventilation.

**Procedure**

*Estimated timing: 2 minutes*

**Assemble the Source Interface**

1. Carefully place the capillary cap over the exposed transfer capillary on the front of the mass spectrometer.

**! CRITICAL STEP** The capillary cap has a small vent hole. Do not cover this hole with your finger during installation, as this will obstruct the vacuum path and may cause a vacuum fault.

1. Position the conical spray shield over the capillary cap, ensuring it is seated correctly on the source mounting plate.

**Mount the ESI Source Door**

1. Align the hinges of the ESI source door with the corresponding mounting points on the instrument and attach the door.
2. Secure the source door in place by firmly closing the locking lever located on the side of the source interface.

**Connect Source Lines**

1. Connect the blue nitrogen gas supply line by pushing it securely into the designated port on the top of the ESI source door.
2. Connect the sample line from the LC system by screwing the finger-tight fitting into the central port on the ESI source door. Tighten until snug, but do not overtighten.

**Expected Results**

- The ESI source is securely mounted to the mass spectrometer.
- The nitrogen gas and sample lines are correctly connected to the source.
- The instrument's vacuum system remains stable, indicating a proper seal has been made.
- The instrument is ready for electrospray operation and analysis.

**Figures**

Figure 1: Installation of the Capillary Cap

[Placeholder for an image showing the gloved hand placing the capillary cap onto the transfer capillary, with an arrow indicating the critical vent hole that must not be obstructed.]

Figure 2: Mounting the ESI Source Door

[Placeholder for an image sequence showing (A) the alignment of the source door hinges with the instrument mount and (B) the securing of the door with the locking lever.]

Figure 3: Final Assembled ESI Source

[Placeholder for an image of the fully installed ESI source, with labels pointing to the connected nitrogen line and sample line.]

**References**

This is a standard instrument operation procedure. Refer to the manufacturer's specific user and maintenance manuals for further details.

#### Selection of example analysis of LLM-as-a-judge

| **Sec-tion** | **Com-pleteness** | **Technical Accuracy** | **Logical Flow** | **Safety** | **Formatting** | **Notes** |
| --- | --- | --- | --- | --- | --- | --- |
| Pro-cedure –  Step 1 | **1** (The AI-generated protocol completely omits the crucial first step of putting on laboratory gloves for contamination control, which is present in the ground truth.) | **4** (The AI's step 1 (placing the capillary cap) is technically accurate, but it's out of sequence as the ground truth prioritizes contamination control first.) | **2** (The omission of the initial PPE step disrupts the logical flow of preparing for the procedure, as contamination control should precede handling components.) | **1** (The AI misses the explicit safety/contamination control step of wearing gloves, which is a significant omission for laboratory work.) | **5** (Both use numbered lists correctly.) | AI's step 1 corresponds to GT's step 2, while GT's step 1 (gloves) is missing from AI. |
| Pro-cedure –  Step 3 | **5** (Both protocols provide complete and equivalent instructions for placing the spray shield.) | **5** (Both instructions are technically accurate.) | **5** (Both follow the same logical sequence for this step.) | **5** (N/A.) | **5** (Both use numbered lists correctly.) | AI's step 2 corresponds to GT's step 3. |

#### Example summary rating of LLM-as-a-judge

- Completeness: 4
- Technical Accuracy: 4
- Logical Flow: 4
- Safety: 3
- Formatting: 5
- Overall (average of the other 5 criteria): 4.0

### Benchmarking of error detection

Using the ten protocols, we generated a benchmark dataset of 28 videos in which an expert scientist performed the procedures. Most videos contained intentionally introduced errors, while some were error-free controls. In total, the dataset comprised 421 steps, including 70 deliberate errors. To mitigate the risk of out-of-distribution errors, these 70 errors were curated by expert scientists to mimic common, real-world procedural mistakes observed in our laboratory such as omitted steps or incorrect step execution. While the resulting error frequency of 17% in this dataset is intentionally higher than one would expect from a trained researcher (to facilitate robust model testing), the error types and proportion are representative of those that occur in practice.

To evaluate performance, we compared the AI-generated lab notes against manually annotated ground-truth notes for each video. These ground-truth notes specified whether each step was correct or incorrect. Every step of the AI-generated lab notes was classified as a true positive, true negative, false positive, or false negative, which formed the basis for calculating accuracy, precision, and recall. For a quantitative analysis of the agent's strengths and limitations, errors were also categorized by their nature (omission, procedural error, added step, deviation of step order) and the perceptual skill required for detection (lab knowledge, proteomics knowledge, spatial resolution, spatial recognition, fast action recognition). This allowed us to calculate recall for each sub-category.

#### Example agent workflow video

<https://zenodo.org/records/17710382/files/ErrorDetection_LabNoteGeneration.mp4?download=1>

#### Example lab note video

<https://zenodo.org/records/17710382/files/ConnectingESISource_forLabNoteGeneration.MP4?download=1>

#### Example video analysis of agent

#### **Table: Chronological Log of Observed Actions**

| **Timestamp** | **Visual/Audio Action** |
| --- | --- |
| 00:00:00 | The video starts, showing the front of the Bruker timsTOF SCP with the ion source interface exposed. The researcher is already wearing white laboratory gloves. |
| 00:00:02 | The researcher's left hand brings the conical, metallic spray shield into view. |
| 00:00:05 | The researcher aligns the spray shield with the central solvation stage on the instrument interface. |
| 00:00:06 | The spray shield is pushed firmly into place. |
| 00:00:10 | The researcher picks up the black, spherical ESI source door. |
| 00:00:13 | The ESI source door is aligned with the hinge on the instrument chassis. |
| 00:00:14 | The door is swung closed, and the metal locking latch is immediately fastened. |
| 00:00:17 | The researcher takes the red sample capillary line, which is connected to a syringe. |
| 00:00:18 | The sample line fitting is connected to the upper inlet port on the ESI source. |
| 00:00:22 | The connection is complete. The researcher's hands move out of frame. |
| 00:00:25 | The video ends. |

#### **Table: Protocol-to-Video Comparison**

| **Step number** | **Step Description** | **Timestamp in 'Video to evaluate'** | **Comparison Result** | **Notes** |
| --- | --- | --- | --- | --- |
| 1 | Put on a fresh pair of laboratory gloves. | 00:00:00 | Aligned | The researcher was already wearing gloves at the start of the video, indicating this step was completed prior to the recorded procedure. |
| 2 | Carefully align the capillary cap with the glass capillary... Gently press it into place. | N/A | ❌ **Omitted:** | This action was not observed. The video begins after the point where this step would have been performed. The capillary cap is presumed to be pre-installed. |
| 3 | Place the conical spray shield over the installed capillary cap. | 00:00:05 - 00:00:06 | ⚠️ **Deviation: Altered step order** | This was the first action performed in the video. The protocol specifies this step should occur after installing the capillary cap (Step 2). |
| 4 | Pick up the spherical ESI source door and align its hinge. | 00:00:10 - 00:00:13 | Aligned | The action was performed exactly as described. |
| 5 | Swing the source door into its closed position. | 00:00:14 | Aligned | The action was performed exactly as described. |
| 6 | Secure the door by fastening the metal locking latch. | 00:00:14 | Aligned | The action was performed immediately after closing the door, as expected. |
| 7 | Connect the nitrogen gas line. | N/A | ❌ **Omitted:** | The transparent nitrogen gas line is visible but was not connected to the ESI source door during the observation. The corresponding inlet port on the source remains empty. |
| 8 | Connect the sample capillary line. | 00:00:17 - 00:00:21 | Aligned | The red sample capillary line was connected to its designated port on the ESI source. |

#### Example ai-generated lab note

**Installation of the ESI Source on a Bruker timsTOF SCP**

**Aim**

This report documents the observed procedure for the installation of an Electrospray Ionization (ESI) source onto a Bruker timsTOF SCP mass spectrometer.

**Materials**

**Equipment**

- Bruker timsTOF SCP Mass Spectrometer
- ESI Source
- Capillary Cap
- Spray Shield
- Personal Protective Equipment (PPE): laboratory gloves
- Syringe filled with sample

**Reagents**

- Nitrogen gas supply (instrument-grade)

**Procedure**

***Timing:****The observed procedure was completed in approximately 22 seconds.*

**Assemble the Ion Source Interface**

1. A fresh pair of laboratory gloves was already being worn at the commencement of the observation (00:00:00).
2. ❌ **Omitted:** The installation of the capillary cap was not performed during the observation. The component was presumably pre-installed on the instrument interface.
3. ⚠️ **Deviation: Altered step order** The conical spray shield was placed over the pre-installed capillary cap and seated correctly on the solvation stage (00:00:05). This was the first observed step in the assembly.

**Mount the ESI Source Door**

1. The spherical ESI source door was picked up, and its hinge was aligned with the corresponding mounting point on the mass spectrometer's chassis (00:00:10 - 00:00:13).
2. The source door was swung into its closed position against the interface (00:00:14).
3. The door was secured by fastening the metal locking latch until it was firmly engaged (00:00:14).

**Connect Gas and Sample Lines**

1. ❌ **Omitted:** The nitrogen gas line was not connected to the designated gas inlet port on the ESI source.
2. The sample capillary line, which was connected to a syringe, was attached to the sample inlet port on the ESI source (00:00:17 - 00:00:21).

**Results**

- The ESI source was securely mounted on the timsTOF SCP instrument front.
- The spray shield and source door were correctly aligned and seated. The capillary cap was presumed to be correctly seated from a prior, unobserved action.
- The sample line was firmly connected to its respective port. However, the nitrogen gas line was not connected.
- Due to the omission of the nitrogen gas line connection, the instrument was not ready for operation as per the protocol's expected outcome. A stable vacuum and operational readiness could not be confirmed.

#### Example evaluation of error detection

| **Step** | **Bench-mark** | **Class** | **Skill** | **AI Re-sponse** | **AI Class** | **Identi-fication** | **Classi-fication** |
| --- | --- | --- | --- | --- | --- | --- | --- |
| 1 | No Error | N/A | N/A | No Error | N/A | No Error (Correctly Identified) | N/A |
| 2 | Error | Omitted | ProteomicsKnowledge | Error | Omitted | Error (Correctly Identified) | correct |
| 3 | No Error | N/A | N/A | Error | Deviation | False Positive | N/A |
| 4 | No Error | N/A | N/A | No Error | N/A | No Error (Correctly Identified) | N/A |
| 5 | No Error | N/A | N/A | No Error | N/A | No Error (Correctly Identified) | N/A |
| 6 | No Error | N/A | N/A | No Error | N/A | No Error (Correctly Identified) | N/A |
| 7 | Error | Omitted | ProteomicsKnowledge | Error | Omitted | Error (Correctly Identified) | correct |
| 8 | No Error | N/A | N/A | No Error | N/A | No Error (Correctly Identified) | N/A |

# References

Skowronek P, Wallmann G, Wahle M, Willems S & Mann M (2025) An accessible workflow for high-sensitivity proteomics using parallel accumulation–serial fragmentation (PASEF). *Nat Protoc* 20: 1700–1729

Zheng L, Chiang W-L, Sheng Y, Zhuang S, Wu Z, Zhuang Y, Lin Z, Li Z, Li D, Xing EP, *et al* (2023) Judging LLM-as-a-Judge with MT-Bench and Chatbot Arena. doi:10.48550/arXiv.2306.05685 [PREPRINT]
